# RETRACTION: Evaluating the Impact of Recombinant Human Epidermal Growth Factor on Scar Formation in Oral and Maxillofacial Traumatic Wound Healing

**DOI:** 10.1111/iwj.70496

**Published:** 2025-04-02

## Abstract

**RETRACTION:** ZhuB. , TongG. , GaoP. , and YanM. , “Evaluating the Impact of Recombinant Human Epidermal Growth Factor on Scar Formation in Oral and Maxillofacial Traumatic Wound Healing,” International Wound Journal 21, no. 4 (2024): e14851, 10.1111/iwj.14851.38563121 PMC10985544

The above article, published online on 02 April 2024, in Wiley Online Library (http://onlinelibrary.wiley.com/), has been retracted by agreement between the journal Editor in Chief, Professor Keith Harding; and John Wiley & Sons Ltd. Following an investigation by the publisher, all parties have concluded that this article was accepted solely on the basis of a compromised peer review process. The editors have therefore decided to retract the article. The authors did not respond to our notice regarding the retraction.

**RETRACTION:** B. Zhu , G. Tong , P. Gao , and M. Yan , “Evaluating the Impact of Recombinant Human Epidermal Growth Factor on Scar Formation in Oral and Maxillofacial Traumatic Wound Healing,” International Wound Journal 21, no. 4 (2024): e14851, 10.1111/iwj.14851.38563121 PMC10985544

The above article, published online on 02 April 2024, in Wiley Online Library (http://onlinelibrary.wiley.com/), has been retracted by agreement between the journal Editor in Chief, Professor Keith Harding; and John Wiley & Sons Ltd. Following an investigation by the publisher, all parties have concluded that this article was accepted solely on the basis of a compromised peer review process. The editors have therefore decided to retract the article. The authors did not respond to our notice regarding the retraction.

